# Perioperative Treatments in Pleural Mesothelioma: State of the Art and Future Directions

**DOI:** 10.3390/cancers17193199

**Published:** 2025-09-30

**Authors:** Luigi Giovanni Cecchi, Marta Aliprandi, Fabio De Vincenzo, Matteo Perrino, Nadia Cordua, Federica Borea, Alessandro Bertocchi, Antonio Federico, Giuseppe Marulli, Armando Santoro, Giovanni Luca Ceresoli, Paolo Andrea Zucali

**Affiliations:** 1 Department of Biomedical Sciences, Humanitas University, Via Rita Levi Montalcini 4, Pieve Emanuele, 20072 Milan, Italy; luigi.cecchi@humanitas.it (L.G.C.); alessandrp.bertocchi@cancercenter.humanitas.it (A.B.); antonio.federico@cancercenter.humanitas.it (A.F.); giuseppe.marulli@hunimed.eu (G.M.); armando.santoro@cancercenter.humanitas.it (A.S.); paolo.zucali@hunimed.eu (P.A.Z.); 2 Department of Oncology IRCCS, Humanitas Research Hospital, Via Manzoni 56, Rozzano, 20089 Milan, Italy; fabio.de_vincenzo@cancercenter.humanitas.it (F.D.V.); matteo.perrino@humanitas.it (M.P.); nadia.cordua@cancercenter.humanitas.it (N.C.); 3 Department of Oncology, Cernusco sul Naviglio, ASST Melegnano e Martesana, 20063 Milan, Italy; federica.borea@asst-melegnano-martesana.it; 4 Division of Thoracic Surgery, IRCCS Humanitas Research Hospital, Via Manzoni 56, Rozzano, 20089 Milan, Italy; 5 Department of Medical Oncology, Humanitas Gavazzeni, 24125 Bergamo, Italy

**Keywords:** pleural mesothelioma, peri-operative treatments, thoracic neoplasms

## Abstract

Pleural Mesothelioma (PM) remains a challenging malignancy associated with asbestos exposure and characterized by poor prognosis. This review aims to consolidate recent findings on the efficacy of perioperative therapies encompassing chemotherapy, surgery, and emerging immunotherapy strategies. Current management strategies debate the role of surgery in early-stage patients, particularly due to the limited success of solitary treatment modalities and significant rates of postoperative complications. Retrospective studies indicate that multimodal treatment, incorporating surgical resection with perioperative chemotherapy, can enhance overall survival (OS), especially in favorable prognostic subsets. However, significant randomized trials, notably the MARS and MARS 2 trials, revealed that the addition of aggressive surgical strategies like extrapleural pneumonectomy (EPP) did not confer survival benefits and was accompanied by heightened morbidity. In light of persistent challenges, integrating perioperative chemotherapy—primarily with platinum-based regimens—has shown improved disease control outcomes. Neoadjuvant chemotherapy permits real-time assessment of tumor responsiveness, providing valuable clinical insights for surgical candidacy. The role of immunotherapy, particularly immune checkpoint inhibitors (ICIs), is also under active exploration, with preliminary results suggesting promising activity and manageable safety profiles. In conclusion, while current protocols primarily recommend surgery for a select group of patients, ongoing investigations into neoadjuvant approaches, adjuvant therapies, and novel immunotherapeutic strategies are crucial for developing effective, personalized treatment paradigms for PM. Future efforts should prioritize clinical trials that integrate these therapies within a structured multidisciplinary approach to optimize patient outcomes.

## Simple Summary

Pleural mesothelioma (PM) is a rare, asbestos-related malignancy with poor prognosis and complex management. The role of surgery remains controversial, though it may benefit select early-stage patients with favorable prognostic features, such as epithelioid histology and absence of nodal or chest wall involvement. International guidelines recommend surgical consideration for patients with good performance status and adequate cardiopulmonary reserve. However, outcomes from the MARS and MARS 2 trials questioned the value of surgery, reporting no survival advantage and higher morbidity, particularly in low-volume centers with limited surgical expertise. In contrast, the EORTC 1205 trial, conducted in specialized centers, demonstrated improved survival and lower operative mortality, underscoring the importance of surgical proficiency. Given the diffuse nature of PM and high recurrence rates, surgery alone is insufficient, necessitating multimodal approaches incorporating chemotherapy and radiotherapy. Emerging evidence also supports integrating immunotherapy, including immune checkpoint inhibitors, as a perioperative strategy to improve long-term outcomes.

## 1. Introduction

Pleural mesothelioma (PM) is a rare and aggressive tumor primarily associated with asbestos exposure, characterized by a poor prognosis [1]. The management of this malignancy remains a subject of ongoing debate. Specifically, while surgical intervention’s role in the treatment of PM is contentious, it has been considered for patients presenting with early-stage disease. Retrospective studies indicate that surgical approaches, when applied within a multimodal treatment framework, may confer advantages over chemotherapy alone, particularly in patients exhibiting favorable prognostic factors, including age below 70 years, epithelioid histological subtype, absence of chest wall infiltration, and no involvement of lymph nodes. The median survival for these cohorts ranges from 17 to 35 months [2].

Current international guidelines recommend surgical options for a select group of patients, particularly those diagnosed with epithelioid PM at an early clinical stage according to the 9th Edition Tumor, Node, Metastasis (TNM) staging system (cT1-3, N0-1, M0; stages I-IIIa), who are in good performance status and possess adequate cardiopulmonary reserve [3,4,5].

However, recent randomized trials, namely, the Mesothelioma and Radical Surgery (MARS) trial and its sequel, the MARS 2 trial, have presented evidence suggesting that the addition of surgical intervention to chemotherapy may be detrimental compared to chemotherapy alone. Notably, the MARS trial involved numerous centers lacking specialized expertise in performing extrapleural pneumonectomy (EPP). Similarly, the MARS 2 trial documented that 45% of surgical cases were conducted at low-volume centers, potentially jeopardizing overall survival (OS) outcomes for patients in the pleurectomy/decortication (P/D) cohort. Expert surgical proficiency is therefore paramount. Conversely, in the MARS 2 trial, the threshold for determining surgical competency was deemed completion of merely five extended pleurectomy decortications [6,7].

Recently published findings from the prospective, randomized phase II EORTC 1205 trial shed new light on the surgical component of PM management [8]. Unlike MARS 2, all surgeries included in EORTC 1205 were conducted exclusively at specialized centers by experienced surgeons trained in the extended P/D procedure. Even though intertrial comparisons may introduce bias and were not the primary focus of the EORTC 1205 trial, the median OS observed within the neoadjuvant chemotherapy arm of EORTC 1205 was greater than 33 months, in stark contrast to the below 24 months reported for resected patients in the MARS 2 trial. Notably, operative mortality at 90 days was recorded at 8.9% in the MARS 2 trial, compared to just 1.7% within the EORTC 1205 cohort [9].

The diffuse growth patterns of PM and the lack of well-defined surgical margins often preclude complete microscopic resection, limiting surgical outcomes to cytoreduction only [10]. Residual microscopic disease is linked to local recurrence in over 75% of cases within one year post-resection [11]. In addition, the risk of extra-thoracic metastasis poses significant challenges in disease management, adversely impacting overall survival rates. Thus, the integration of surgical approaches within a multimodal treatment paradigm—including perioperative chemotherapy and radiotherapy—aims to improve disease control and survival rates. Multiple retrospective analyses affirm that PM patients receiving adjunctive chemotherapy and/or radiotherapy alongside radical-intent surgery experience significantly improved OS compared to those undergoing surgery alone [12,13,14,15]. Moreover, several phase II prospective trials highlight the feasibility and favorable survival outcomes associated with trimodal approaches, especially in patients able to complete the entire therapeutic regimen, despite completion rates falling below 50%, primarily due to a high incidence of adverse events (AEs) [16,17,18].

The optimal timing and combination of surgical intervention, chemotherapy, and radiation require further elucidation. Given the promising efficacy of immunotherapy—both standalone and in combination with chemotherapy—in the first-line setting, immune checkpoint inhibitors (ICIs) and other innovative immunotherapeutic strategies, such as cancer vaccines and dendritic cell therapy, are under investigation for their potential role in the perioperative management of PM.

This review aims to provide a comprehensive overview of the current landscape and future directions for perioperative therapies in PM, encompassing perioperative radiotherapy, chemotherapy, intraoperative treatments (e.g., hyperthermic chemotherapy), and perioperative immunotherapy.

## 2. Search Strategy

A systematic literature search was conducted utilizing the PubMed database, employing search terms including “mesothelioma,” “perioperative therapy,” “neoadjuvant chemotherapy,” “adjuvant chemotherapy,” “neoadjuvant radiotherapy,” and “adjuvant radiotherapy”. Additionally, we examined reference lists from pertinent abstracts and publications presented at conferences held by the International Association for the Study of Lung Cancer (IASLC), the International Mesothelioma Interest Group (IMIG), the American Society of Clinical Oncology (ASCO), and the European Society for Medical Oncology (ESMO).

## 3. The Role of Radiotherapy

Radiotherapy (RT) as part of the multimodality treatment of PM is used to reduce the risk of local relapse after macroscopically radical surgery. Table 1 summarizes the main trials evaluating RT in perioperative setting.

Initial phase II clinical trials show that RT is associated with a median PFS of 8–14 months and a median OS of 15–29 months, alongside locoregional relapse rates of 12–16% [16,19,20]. A significant advancement was demonstrated in a randomized phase III study that enrolled patients with gross residual disease post non-radical lung-sparing surgery [21]. In this trial, patients randomized to receive radical hemithoracic radiotherapy (RHR) at 50 Gy in 25 fractions (with a 60 Gy boost to the gross tumor volume) exhibited a two-year overall survival rate of 58%, compared to 28% in patients receiving palliative radiotherapy (PR). Additionally, local control improved significantly, with a two-year local relapse rate of 27.1% versus 82.7% for PR.

Despite these promising results, the overall efficacy of RT in large randomized clinical trials has been inconsistent, primarily due to the rarity of PM, low patient accrual in prospective studies, and the resultant low statistical power of findings. A propensity score-matched analysis using the Surveillance, Epidemiology, and End Results (SEER) database highlighted a survival benefit associated with radiotherapy, demonstrating a 32% reduction in the risk of death when RT was part of the treatment regimen [22,23]. Similar data from the National Cancer Database also indicated improved survival in stage I–II PM patients treated with RT [24]. Retrospective studies have shown low locoregional recurrence rates among patients receiving RT, with two-year PFS rates of 30–40% and local control rates of about 60% [25,26]. However, a recent propensity score-matched analysis indicated no difference in time-to-ipsilateral recurrence between patients receiving adjuvant RT and those who did not [27].

Given these mixed findings, international guidelines continue to recommend RT based on the body of evidence from phase II clinical trials and observational studies [3,5]. However, the management of PM with RT carries a high risk of treatment-related toxicity due to the extensive irradiated fields and proximity to radiosensitive organs. Implementing strict dosimetric constraints and utilizing varying therapeutic sequences (neoadjuvant vs. adjuvant) has helped improve the safety profile of RT [28,29]. Still, complications remain prevalent, with reported rates of grade 3–5 adverse events like pneumonitis (5–10%), dermatitis (8–17%), and esophagitis (10–15%). Critical pulmonary complications, particularly in patients undergoing extensive surgical interventions, have also been observed [21,25,26].

RT’s role has been assessed in conjunction with different surgical techniques, particularly extrapleural pneumonectomy (EPP). The SAKK17/04 phase II trial sought to establish the benefits of adjuvant RT following neoadjuvant chemotherapy and EPP. However, due to slow accrual, the study was prematurely halted after randomizing only 54 patients, with 23 receiving adjuvant RT. The trial revealed no significant advantage in locoregional recurrence-free survival (RFS) between the observation and RT groups [30,31]. Despite the lung being absent in the irradiated hemithorax, adjuvant RT after EPP remains associated with a considerable risk of pulmonary toxicity due to low functional reserve, necessitating stringent dosimetric planning [29].

Preoperative RT has also been explored with the SMART trial, wherein patients received a total of 25 Gy over five fractions prior to EPP to minimize tumor spillage and local recurrence [32]. This approach resulted in a median disease-free survival of 18.0 months and a 5-year local relapse rate of 20.1%, though it revealed a high toxicity rate, with 49% experiencing grade 3–4 adverse effects.

Conversely, MARS trial results indicated no survival benefit from the use of EPP, alongside elevated morbidity. A propensity score analysis further illustrated the lack of improved oncological outcomes from EPP compared to pleurectomy/decortication (P/D) [14].

The IMPRINT trial evaluated the role of adjuvant RT following chemotherapy and P/D. This study delivered a total of 50.4 Gy in 1.8 Gy fractions without planned boost doses, reporting a 7% incidence of grade 3 radiation pneumonitis. The median PFS post-treatment was 12.4 months, with approximately 55% of patients experiencing local recurrences as the first site of disease relapse [33].

Considering these findings, multimodal treatment involving P/D followed by adjuvant RT is recommended for resectable PM. However, due to its associated toxicities, such treatments should be administered in specialized centers with experience in PM management. Multidisciplinary discussions are advised to optimize RT timing [3,5].

Several ongoing trials are exploring the feasibility of RT in the neoadjuvant context, including the SMARTER trial (NCT04028570), which aims to assess dose levels in PM patients receiving background and boost radiation to bulky disease sites [34]. Another trial, SMARTEST (NCT05380713), investigates combining low-dose cyclophosphamide with sub-ablative RT, followed by consolidation with tremelimumab and durvalumab [35].

Advancements in hypofractionated RT regimens, showing promise in other disease contexts, have instigated their evaluation in PM. The MESO-RT trial (NCT03269227) studies adjuvant accelerated hypofractionation (30 Gy in 5 fractions) after P/D, yielding preliminary results with a time-to-progression of 26.9 months and no grade 3–4 adverse events recorded [36,37].

Emerging proton therapy techniques also show potential in PM treatment due to the Bragg Peak property, allowing for targeted high-energy doses minimizing exit dose to adjacent organs. However, challenges remain regarding organ motion and the uncertainties surrounding dose delivery [38,39]. Therefore, while RT in PM treatment offers potential benefits, further validation through larger, controlled studies is essential to confirm its efficacy and safety. Ongoing and emerging trials may pave the way for enhanced treatment strategies, ultimately improving patient outcomes [40,41,42].

**Table 1:** Main trials evaluating radiotherapy in perioperative setting.

Study Reference	Design	Radiation Dose	Intervention	Patients N°	Primary Endpoint	Secondary Endpoint
**Neoadjuvant**						
**SMART trial** (2021) [32]	Single center, single arm phase II	25 Gy/5 Fr + 5 Gy boost to high risk areas	Neoadjuvant ipsilateral hemithorax RT followed by EPP in resectable PM	96	30-day G3-4 complication rate (presp. < 35%)—49%	5-year local recurrence 20.1% [11.4–28.8]; 5-year distant recurrence 63.3% [52.3–74.4]; mOS 24.4 mo [18.5–31.1 mo]; mDFS 18.0 mo [12.6–21.7 mo]
**Adjuvant**						
**Trovo M. et al.** (2021) [21]	Single center, randomized phase III	RHR Arm (50 Gy/25 Fr, GTV boost to 60 Gy); PR Arm (21 Gy/3 Fr. Or 20–30 Gy/5–10 Fr)	Radical hemithoracic RT vs. Palliative RT in non-metastatic PM treated with non-radical lung-sparing surgery and CT	108	mOS: 25.6 mo vs. 12.4 mo (HR 0.54, 0.31–0.95; *p* = 0.031)	mPFS: 9.8 mo vs. 5.4 mo [HR0.15; 0.09–0.27; *p* < 0.001]; median locoregional RFS: 10.1 mo vs. 5.6 mo [HR 0.14; 0.08–0.25, *p* < 0.001]; median distant RFS 12.8 mo vs. 5.5 mo [HR 0.99; 0.54–1.80, *p* = 0.969]
**SAKK 17/04** (2015) [30]	Multicenter, randomized, phase II	Part 2–3 schedules: 45 Gy/25 Fr (boost 57.6 Gy); 46 Gy/23 Fr (boost 56 Gy); 45.5 Gy/26 Fr (boost 55.9 Gy)	Adjuvant high-dose RT vs. observation in patients who achieved macroscopic surgical resection after neoadjuvant CT.	Part 2–54	Part 2—Locoregional RFS—9.4 mo [6.5–11.9] vs. 7.6 mo [4.5–10.7]	mRFS—7.6 mo [5.2–10.6] vs. 5.7 mo [3.5–8.8]; mOS—19.3 mo [11.5–21.8] vs. 20.8 mo [14.4–27.8]; feasibility: 83%; QoL (no differences)
**IMPRINT** (2016) [33]	Two-center phase II	50.4 Gy/28 Fr	Adjuvant RT after neoadjuvant cisplatin-pemtrexed chemotherapy and P/D	27	Incidence of G3 RP: 7.4% (2/27)	mPFS: 12.4 mo; mOS: 23.7 mo

CT: Chemotherapy; mDFS: median Disease-Free Survival; EPP: Extrapleural Pneumonectomy; Fr: Fraction; Gy: Gray; PM: Pleural Mesothelioma; mo: months; mOS: Median Overall Survival; Presp: prespecified; mPFS: Progression-Free Survival; PR: Palliative Radiotherapy; QoL: Quality of Life; RFS: Relapse-Free Survival; RHR: Radical Hemithoracic Radiotherapy; RP: Radiation Pneumonitis; RT: Radiotherapy.

## 4. The Role of Chemotherapy

Chemotherapy has played a pivotal role in the management of PM, particularly in perioperative settings. Initial studies focused on the efficacy of platinum-based compounds, such as carboplatin and cisplatin, in conjunction with gemcitabine. The phase III EMPHACIS study (2003) revealed that the combination of cisplatin and pemetrexed significantly enhanced response rates (RR), PFS, and OS compared to cisplatin alone. Consequently, this regimen has become the new standard of care in both first-line and perioperative settings for patients with unresectable PM [3,4,5,6]. For many years, chemotherapy was predominantly administered as adjuvant treatment post-surgery, but the integration of neoadjuvant chemotherapy has become increasingly prevalent. Table 2 presents a summary of key trials examining chemotherapy in perioperative contexts.

### 4.1. Neoadjuvant Setting

#### 4.1.1. Platinum Compound Plus Gemcitabine

One retrospective trial and four prospective trials established the tolerability and efficacy of neoadjuvant chemotherapy using platinum compounds and gemcitabine in trimodal treatment for PM [15,17,45,46]. Notably, a retrospective study by Opitz et al. involving 63 mesothelioma patients indicated an RR of 32% and a significant rate of morbidity (62%) and mortality (3.2%) following three cycles of cisplatin plus gemcitabine before undergoing extrapleural pneumonectomy (EPP) and radiotherapy [45].

A multicentral phase 2 trial, enrolling 61 patients with potentially resectable PM, assessed this combination’s effectiveness further [17]. Results showed a high chemotherapy completion rate (95%) but identified grade 3–4 adverse events (AEs) in 31% of cases. Among the 45 patients who underwent EPP, major postoperative complications occurred in 35%, with a postoperative mortality rate of 2.2%. The median OS for the overall population was 19.8 months, increasing to 23 months for patients who underwent EPP. The study highlighted an acceptable safety profile, consistent with various other prospective trials reporting completion rates ranging from 53% to 95% and an RR of 26–33%.

#### 4.1.2. Platinum Compound Plus Pemetrexed

Several retrospective studies have explored the utility of neoadjuvant platinum-based regimens with agents other than gemcitabine, including pemetrexed [15,26,47,48,49,50]. For instance, research from the Swedish Medical Center Institute on 55 PM patients treated with platinum-based chemotherapy reported a 25-month median OS, emphasizing that both nodal positivity and macroscopically positive margins were associated with worse prognoses [47].

In a study by De Perrot et al. encompassing 60 PM patients, the use of cisplatin combined with vinorelbine or pemetrexed as induction chemotherapy prior to EPP and adjuvant high-dose hemithoracic radiotherapy led to an OS of 14 months for the entire cohort, significantly improving to 59 months for patients without nodal involvement who completed all treatments [48]. Moreover, N2-positive disease, incomplete macroscopic resection and non-epithelioid histology were reported as negative prognostic factors.

Further analyses examined the safety and efficacy of pemetrexed in conjunction with carboplatin or cisplatin. For example, a retrospective comparison by Pasello et al. found that patients receiving carboplatin had a longer OS and lower postoperative mortality than those on cisplatin [49]. These findings suggest that optimizing drug selection based on toxicity profiles may enhance treatment outcomes, warranting validation in randomized trials.

In a retrospective cohort study involving 251 patients treated with EPP following induction chemotherapy (161 of whom received platinum/pemetrexed), major morbidity and mortality rates were documented at 30% and 5%, respectively, reflecting that well-selected patients at high-volume centers can tolerate these procedures relatively well [50].

Additional prospective studies reinforced the efficacy of neoadjuvant pemetrexed and platinum in patients with localized PM, demonstrating completion rates of 93–96% and OS ranging from 15.5 to 19.9 months following trimodal therapy [15,16,19,20,25,51]. In the multicentre phase II SAKK 17/04 trial, 151 patients were enrolled and received neoadjuvant chemotherapy, of whom 113 (75%) underwent EPP [30]. The radiological ORR to chemotherapy was 34%. Among 113 patients who underwent EPP, the macroscopic complete resection (MCR), the primary endpoint of the study, was achieved in 64% of cases. The post-operative mortality rate was 8.8%. The median OS in the overall population enrolled in part 1 was 15 months. Patients with N0-1 stage and epithelioid histology achieved a median OS of 18.5 compared to 10.8 months of patients with N2 disease and biphasic or sarcomatoid subtypes.

### 4.2. Adjuvant Setting

Chemotherapy’s role extends to the adjuvant context, aiming to reduce recurrence risk post-surgery without delaying operations. However, administering chemotherapy afterwards complicates response evaluation and patient selection based on disease aggressiveness.

A retrospective analysis of 183 PM patients who underwent EPP followed by chemotherapy indicated a median OS of 19 months, with notably better outcomes for patients with epithelioid histologies and negative resection margins [52]. After EPP, the perioperative mortality rate was 3.8%, and morbidity was reported in 50% of cases. Investigating smaller cohorts and various chemotherapy regimens, such as doxorubicin plus cyclophosphamide or cisplatin combinations, showed similar trends in treatment tolerability and survival outcomes.

These findings are consistent with those of other retrospective trials supporting the feasibility and acceptable safety profile of adjuvant chemotherapy in the multimodal treatment of selected patients with PM [53,54,55].

Prospective studies, such as Bölükbas et al.’s investigation involving 35 patients receiving adjuvant cisplatin and pemetrexed, exhibited promising results—including a 30-month median OS in those completing the full treatment [43,56,57].

Current guidelines from the NCCN recommend platinum-pemetrexed induction for resectable PM, while the ESMO supports either neoadjuvant or adjuvant chemotherapy [3,5]. The efficacy of such strategies has been debated, with studies not showing significant OS differences between neoadjuvant and adjuvant approaches, emphasizing patient selection and predictive factors like histology and nodal involvement remain crucial [44,58,59,60].

The potential of chemotherapy in both neoadjuvant and adjuvant settings for PM is under extensive investigation. Ongoing trials, summarized in Table 3, aim to elucidate optimal sequences and combinations of treatment to enhance patient outcomes [8,61]. 

In particular, the prospective randomized phase 2 EORTC 1205 trial (NCT02436733) compared the neoadjuvant or adjuvant approaches for chemotherapy in the context of a trimodal treatment [8]. The primary endpoint is the successful completion of planned treatment. In general, 56 patients (81%) completed three cycles of chemotherapy, while 58 patients (84%) underwent surgical procedures. Among the 64 patients included in the primary analysis, 21 patients (70%) in arm A achieved the statistical endpoint for success, compared to 17 (50%) in arm B. The median PFS and OD were 10.8 and 27.1 months for arm A and 8.0 33.8 months for arm B, respectively. Therefore, EORTC 1205 did not successfully identify a preferred sequence for pre-operative or post-operative chemotherapy. Both procedures are feasible with low mortality rates, although they are associated with consistent morbidity.

### 4.3. Intraoperative Chemotherapy

In the context of multimodal treatment of PM, several studies explored the role of Hyperthermic Intraoperative Chemotherapy (HIOC) [73,74,75]. HIOC involves the direct application of chemotherapy to the surgical bed after resection. The rationale behind this procedure is to achieve loco-regional disease control using a high concentration of cytotoxic therapy combined with hyperthermia, which enhances the penetration depth of the drug and directly damages residual tumor cells. It is crucial to underline that HIOC is performed in experienced centres. In fact, perioperative mortality rates reported in the literature are low, while AEs range from 4% to 65%. Mild-to-moderate renal failure and deep vein thrombosis are the most frequent complications [74]. 

Van Sandick et al. retrospectively compared the outcomes of 35 PM patients treated with radical surgery, of which 15 underwent subsequent adjuvant radiotherapy (54 Gy), to the outcomes of 20 patients who received HIOC followed by radiotherapy on surgical scars (24 Gy) [76]. The adjuvant radiotherapy group demonstrated superiority over the HIOC group in terms of OS (29 vs. 11 months, although not statistically significant), median time to local recurrence (not reached vs. 9 months, *p* = 0.003), and postoperative complications (53% vs. 70%).

Another retrospective study enrolled 53 patients with epithelioid/biphasic PM (cT1-3, N0-1, M0) treated with P/D followed by HIOC and 4 cycles of adjuvant cisplatin plus pemetrexed [73]. The most common AE was an increase in serum creatinine (>2 mg/dL) reported in 11% of patients, with 4% requiring renal supportive therapy. The median OS was 52.4 months, and the median DFS was 18.7 months. Similarly, Sugerbaker et al. retrospectively analysed 103 patients treated with cytoreductive surgery, out of which 71 received HIOC. The use of hyperthermic cisplatin (175 to 225 mg/m^2^)was associated with a delayed time to recurrence (27.1 vs. 12.8 months) and an improved survival (35.3 vs. 22.8 months), especially among patients who did not receive hemithoracic radiotherapy and those without nodal involvement (N1/N2) [77]. 

More recently, Elsayed et al. reported the outcomes of a retrospective–prospective trial (*n* = 55) of patients treated with surgery (either P/D or extended P/D) (*n* = 30) or surgery followed by HIOC (cisplatin 125 mg/m^2^ for 70 min) (*n* = 25). Despite the trend towards an improved PFS (8 months [95% CI—4.3–11.6] vs. 6 months [95% CI—2.5–9.9]) and OS (28 months [95% CI—21.5–34.5] vs. 22 months [95% CI—17.5–26.5]) in the HIOC group, the trial failed to reach statistical significance [75,78]. 

To date, despite preliminary data suggesting the potential benefit of including HIOC as part of the multimodal treatment of localized MPM, the current evidence remains limited and largely retrospective, preventing its adoption as a standardized approach [4]. Therefore, the HIOC should be only performed in specialized medical centres in the context of clinical trials.

## 5. The Role of Immunotherapy

The promising results from immunotherapy as first-line treatment for pleural mesothelioma (PM) have generated increasing interest in the application of immune checkpoint inhibitors (ICIs) within perioperative management protocols. The unique immunological characteristics of PM may provide a compelling rationale for the use of ICIs in this context. The tumor microenvironment in PM is complex, comprising 20–80% tumor-infiltrating lymphocytes (TILs), with cytotoxic T cells constituting 5–15%. A significant population of inflammatory cells is made up of tumor-associated macrophages (TAMs), which often exhibit a pro-tumor phenotype. These TAMs contribute to the establishment of an immunosuppressive environment by secreting interleukin-10 (IL-10) and transforming growth factor-β (TGF-β), as well as expressing Programmed Death-Ligand 1 (PD-L1) [79,80]. 

Increased PD-L1 expression in PM is associated with poorer prognostic outcomes. Retrospective analyses indicate that patients with elevated PD-L1 levels and treated solely with chemotherapy or those who underwent macroscopic surgical resection experienced worse survival rates [81,82]. Conversely, patients demonstrating ≥1% PD-L1 expression showed improved survival outcomes when treated with dual ICIs, as reported in the CheckMate 743 trial [83]. Similar promising results have been observed in the IND227 trial, a Phase III study comparing platinum-based chemotherapy plus pembrolizumab versus chemotherapy alone for unresectable PM, particularly in the sarcomatoid subtype, which has historically been excluded from multimodal treatment trials due to its aggressive nature and limited prognosis [84]. Given the association of high PD-L1 expression with the sarcomatoid phenotype and the enhanced effectiveness of immunotherapy in this subgroup, there exists a significant opportunity to explore the implementation of ICIs in the perioperative management of sarcomatoid PM [85].

Multiple clinical trials are presently investigating the use of neoadjuvant and adjuvant ICIs. In the adjuvant setting, the AtezoMeso trial (NCT04996017), a Phase III, double-blind, placebo-controlled study, evaluates the administration of atezolizumab (1200 mg IV every 21 days) to patients who have undergone P/D without measurable macroscopic residual tumor and have completed at least four cycles of perioperative platinum-pemetrexed chemotherapy. Patients are randomly assigned in a 2:1 ratio to receive adjuvant immunotherapy for 12 months, with the primary endpoint being disease-free survival and secondary endpoints focusing on overall survival and quality of life [71,86].

Nivolumab was also under investigation as part of an adjuvant regimen following cytoreductive surgery in the NICITA trial, a Phase II multicentre study targeting stage I-III PM patients who have undergone P/D [87]. Here, patients were randomized to receive adjuvant chemotherapy with or without nivolumab for four cycles, followed by a 12-month maintenance treatment in the experimental group. The primary endpoints included time-to-next treatment and rates of AEs [88].

Pre-operative administration of ICIs has shown improved outcomes in various tumor types, including melanoma and non-small-cell lung cancer [89,90,91]. The rationale for this approach lies in the potential for ICIs to target micrometastatic disease that is undetectable by standard imaging, thereby enhancing the anti-tumor immune response. Several mechanisms amplify the effectiveness of neoadjuvant ICIs, including a higher presence of endogenous antigens and an intact tumor microenvironment in the primary lesion, leading to stronger activation and clonal expansion of anti-tumor T cells upon immune checkpoint blockade.

Research conducted by Blank et al. has demonstrated superior T cell expansion and clonal diversity following neoadjuvant ICI treatment compared to adjuvant administration, highlighting the strategic advantage of preoperative intervention [92].

Several Phase I/II clinical trials are currently underway to assess the efficacy of neoadjuvant ICIs. For instance, a recently published Phase II randomized window of opportunity trial evaluated the effects of anti-PD-L1 (durvalumab), dual checkpoint blockade (durvalumab and tremelimumab), or placebo in a preoperative setting [11]. The primary endpoint of the trial was the evaluation of the changes in the CD4/CD8 ratio in the tumor microenvironment. Among the 24 enrolled patients, those receiving dual therapy exhibited significantly longer overall survival (OS) and disease-free survival compared to monotherapy. In patients who received ICIs, a higher frequency of intra-tumoral CD8+ T cells was observed, while there was no difference in the frequencies of CD4+ T cells, TAMs, and Natural Killer (NK) cells. In patients treated with dual ICIs, an increase in circulating CD57+ T cells was observed, which were enriched for CD8+ and CD4+ effector cells. Collectively, these results highlight the promising effects of even a single-dose administration of ICIs in the pre-operative setting.

Additionally, pembrolizumab is being evaluated in a single-arm Phase I trial (NCT03760575) and in a multi-site phase II trial (NCT06155279; IOV-Me-01-2023-CHIMERA) where patients are scheduled to receive neoadjuvant immunotherapy (combined with platinum-based chemotherapy in the CHIMERA trial) prior to surgery, followed by adjuvant pembrolizumab [64].

The role of atezolizumab is also being explored in a Phase I pilot study (NCT03228537), where patients receive four cycles of cisplatin and pemetrexed alongside atezolizumab, followed by surgery with or without adjuvant radiotherapy and ICI maintenance for 12 months [67]. Preliminary results showed a total of 28 enrolled patients, of whom 64.2% underwent surgical resection, and 53.5% began adjuvant immunotherapy [93].

Two ongoing clinical trials are also evaluating nivolumab in association with platinum-based chemotherapy (NCT04162015) or with ipilimumab as neoadjuvant treatments (NCT03918252) [65,66]. 

In conjunction with ICIs, novel immunotherapeutic strategies, including cancer vaccines and dendritic cell (DC) therapies, are being actively investigated. Notably, cancer vaccines do not rely on pre-existing tumor-T cell priming. They deliver tumor-specific antigens along with adjuvant agents to stimulate DC activation, leading to the expansion of tumor-directed CD4+ T cells and cytotoxic lymphocytes.

A Phase II double-blind, placebo-controlled trial evaluated the efficacy of the Wilms Tumor-1 (WT-1) targeting vaccine galinpepimut-S in WT-1 positive patients post-multimodal treatment [62]. While the study was prematurely halted due to futility, it reported a non-significant trend toward improved PFS and OS in the treatment arm. Additionally, studies are assessing the combination of WT-1 directed vaccines with nivolumab in platinum-refractory PM cases [94].

Moreover, a Phase I/Ib trial (NCT04525859) is examining the safety of the polyinosinic-polycytidylic acid (poly-ICLC) vaccine prior to surgical resection. Preliminary results suggest enhanced antigen presentation and T-cell responses, although safety measures are a primary focus of the ongoing study [95,96]. 

DC therapy, which enables the vaccination of patients with a comprehensive array of tumor antigens to induce T-cell responses, has shown promise in a Phase I trial. In fact, in this trial DC therapy led to meaningful disease control and an extended OS (24 months) in treated patients [97].

Given the encouraging outcomes demonstrated by these novel approaches, the ENSURE trial (NCT05304208) is currently assessing the integration of DC therapy into the neoadjuvant treatment regimen, monitoring outcomes like surgery without delays and PFS [98]. 

Following the success of Chimeric Antigen Receptor (CAR) T-cell therapy in hematological malignancies, its potential application has also been explored in malignant pleural mesothelioma (MPM) [72]. In this context, most receptor designs have targeted the tumor-associated antigen (TAA) mesothelin (MSLN), which is considered an attractive candidate given its limited expression in normal mesothelial cells and marked overexpression in mesothelioma. Importantly, higher mesothelin expression has been associated with more aggressive disease behavior [99]. Several early-phase clinical trials have evaluated CAR-T cells in advanced MPM, with preliminary results suggesting feasibility and early signs of clinical activity [99,100]. Building on these findings, subsequent studies have investigated alternative receptor designs and combination strategies with immune checkpoint inhibitors (ICIs), with the dual aim of enhancing antitumor efficacy and mitigating T-cell exhaustion. To improve tumor homing, intrapleural administration of engineered T cells has also been employed, thereby bypassing some of the barriers to lymphocyte trafficking within the tumor microenvironment [101]. Despite these encouraging developments, CAR-T-based approaches have not yet been tested in the perioperative setting.

Table 3 summarizes the main ongoing trials evaluating new therapeutic strategies in perioperative setting.

## 6. Discussion

Over the past decade, an increasing effort has been focused on intensifying the treatment of resectable PM, with the aims of improving clinical outcomes and reducing the risk of disease relapse.

Macroscopically radical surgery has traditionally been considered a crucial element in the management of resectable PM according to international guidelines, even in the absence of solid prospective evidence supporting this approach [3,4,5]. However, the results of the MARS trial, which evaluated the efficacy of EPP, and the more recent preliminary results of the MARS 2 trial call into question the idea that surgery should be universally considered the standard of care for all patients with resectable PM [6,7]. The difficulty in obtaining a complete microscopic resection renders surgical intervention an insufficient strategy to establish adequate disease control, even if included in a multimodal program. However, surgical expertise is paramount. In contrast to the MARS2 trial, surgeries in the EORTC 1205 study were conducted exclusively at centers of excellence, with all participating surgeons trained in the e-P/D procedure. This led to a longer median overall survival and a lower 90-day operative mortality compared to the MARS2 trial. The MAR2 study did not exclude the use of surgery as part of the integrated treatment of resectable PM. However, the trial results have defined a new challenge in identifying and selecting patients who would benefit from the implementation of surgery in PM. Current criteria, including age, sex, histological type, and stage, have proven insufficient for patient selection, highlighting the need for new biomarkers.

Regarding chemotherapy, the platinum-based doublet, administered before or after surgery, represents a crucial element in the treatment strategy for PM, especially considering the results of the MARS2 study [8]. The optimal timing for the administration of perioperative treatment is still being evaluated. Small prospective studies or retrospective analyses observed that both treatment sequences are associated with satisfactory completion rates and AE rates [16,17,30,53,56,57]. Recently, the EORTC 1205 trial did not successfully identify a preferred sequence for pre-operative or post-operative chemotherapy: both procedures are feasible with low mortality rates, although they are associated with consistent morbidity [8]. In general, neoadjuvant chemotherapy has not been associated with an increased risk of surgical complications. Furthermore, the study by Marulli et al. demonstrated improved lung function indices among patients who received induction chemotherapy, especially among those who achieved a disease response [44]. Another advantage of neoadjuvant chemotherapy is its ability to provide an in vivo assessment of the disease’s chemo-sensitivity. Krug et al. have also described the prognostic role of radiological disease response in patients undergoing neoadjuvant chemotherapy. If validated in prospective clinical studies, the radiological response may be included in the selection criteria for patients to be candidates for surgery. On the other hand, evidence regarding adjuvant chemotherapy comes primarily from retrospective data, showing favourable completion rates and survival outcomes consistent with the preoperative setting [56,57]. The main concern for adjuvant treatment is represented by the delay caused by surgery-related morbidity, especially in cases of more invasive approaches such as EPP.

Considering the results of the EORTC 1205 study, the decision on each individual case should be based on the careful evaluation of various factors, including the disease extension, the type of surgical intervention, and the risk factors for postoperative morbidity. It is notable that, among the surgical community, there is a general agreement that P/D should be privileged over EPP when technically feasible, as results are comparable in term of DFS and OS but with significantly lower morbidity and mortality, thus leaving more chance for adjuvant treatments and improving quality of life [102].

Similar to surgery, radiotherapy has undergone a considerable evolution in the treatment of resectable PM. Neoadjuvant and adjuvant RT has shown considerable clinical outcomes in selected patients treated with EPP [30,32] and is currently being studied as part of perioperative treatment with P/D [33,34,35]. Even if aimed at preventing tumor escape or eradicating microscopic residual disease, RT is still associated with a severe toxicity profile, requiring strict dosimetric constraints and careful patient selection. The emerging proton-based technology could offer a more selective and safe approach, although its prospective validation is still pending [40].

Finally, considering the results of the CheckMate743 and IND227 studies, several ongoing trials are currently investigating perioperative strategies involving ICIs [11,64,65,66,67,71,84,87,88]. The findings of early-phase trials confirm the feasibility of ICI combinations in PM as well as their favourable toxicity profile [11]. Similar to other tumor types, the results of these investigations may provide evidence on the optimal application of ICI in various settings of resectable PM.

Regardless of the contribution of individual therapies within the treatment program, determining the therapeutic approach for each case of resectable PM requires a comprehensive evaluation of both the patient’s clinical profile and disease characteristics. As recommended by the latest international guidelines, the therapeutic decision-making should take place within high-volume centres and involve a multidisciplinary team composed of experts in PM. This is the only way to guarantee the patient the most effective therapeutic strategy for the control of their disease while limiting treatment-related toxicity [3,5].

## 7. Conclusions

Multimodal perioperative treatments may provide a survival benefit in selected patients and in high-volume specialized centers, yet it is limited by high morbidity. Future directions should focus on the identification of optimal perioperative treatments, as well as refine patient selection, with the aims of reducing the risk of disease relapse and maximizing survival outcomes.

## Figures and Tables

**Table 2:** Main trials evaluating chemotherapy in perioperative setting.

Study Reference	Design	Agent	Intervention	Patients N°	Primary Endpoint	Secondary Endpoint
**Neoadjuvant chemotherapy**						
**ISRCTN95583524** (MARS) (2011) [6]	multicenter randomized (1:1) controlled feasibility trial	platinum-based CT	Prerandomisation registration phase induction platinum-based CT followed by clinical review. After further consent, randomization (1:1) to: Arm A EPP followed by postoperative hemithorax RT; Arm B no EPP.	Pre-randomization registration phase: 112; Randomization phase 50: Arm A n° 24, Arm B n° 26	feasibility of randomly assigning 50 patients in 1 year: not reached 50 patients randomized in 3 years	Arm A: n° of patients completing EPP: 16 (66.7%). Post-operative mortality rate: 12.5%. Median OS: 14.4 months. ArmB: Median OS: 19.5 months. HR 1.90 (95% CI 0.92–3.93)
**Weder et al.** (2007) [17]	phase 2 single-arm multicenter trial	cisplatin plus gemcitabine	Neoadjuvant cisplatin + gemcitabine, EPP and post-operative RT	61	QoL: no significant deterioration reporte	CT completion rate: 95%. N° of patients completing EPP: 45 (74%). MCR rate: 61%. Post-operative mortality rate: 2.2%. Median OS ITT population: 19.8 months. Median OS in patients undergoing EPP: 23 months.
**Krug et al.** (2009) [16]	phase 2 single-arm multicenter trial	cisplatin plus pemetrexed	Neoadjuvant cisplatin + pemetrexed, EPP and post-operative hemithoracic RT	77	pCR rate: 5.3%	CT completion rate: 83%. Radiological RR: 32.5%. N° of patients completing EPP: 54 (70%). Post-operative mortality rate: 4%. Median PFS ITT population: 10.1 months. median OS ITT population: 16.8 months. Median OS in patients undergoing EPP: 21.9 months.
**EORTC 08031** (2010) [19]	phase 2 single-arm multicenter trial	cisplatin plus pemetrexed	Neoadjuvant cisplatin + pemetrexed, EPP and post-operative RT	58	Success of treatment rate: 42.1%	CT completion rate: 93%. N° of patients completing EPP: 42 (74%). Post-operative mortality rate: 6.5%. Median PFS ITT population: 13.9 months. Median OS ITT population: 18.4 months. Median OS in patients completing trimodality therapy: 37 months.
**Rea et al.** (2013) [20]	phase 2 single-arm, open label trial	cisplatin plus pemetrexed	Neoadjuvant cisplatin + pemetrexed, EPP and post-operative hemithoracic RT	54	median EFS: 6.9 months	CT completion rate: 96.3%. N° of patients completing EPP: 45 (83.3%). Post-operative mortality rate: 4.4%. Median PFS ITT population: 8.6 months. Median TTR: 4.8 months. Median OS ITT population: 15.5 months.
**Adjuvant chemotherapy**						
**Pagan et al.** (2006) [43]	single-arm, open label trial	Paclitaxel plus carboplatin	EPP, adjuvant paclitaxel + carboplatin and RT	54	OS 5-year rate in survived patients after EPP: 19%.	N° of patients completing EPP: 44 (81%). CT completion rate: 73%. Post-operative mortality rate: 4.5%. Median OS in survived patients after EPP: 20 months.
**Lang-Lazdunski et al.** (2012) [44]	Nonrandomized single-center trial	Cisplatin plus gemcitabine/ pemetrexed	Arm A: neoadjuvant CT, EPP and post-operative RT. Arm B: P/D and HIOC, followed by prophylactic RT and adjuvant CT	Arm A: 25; Arm B: 54	Arm A median OS: 12.8 months; Arm B Median OS: 23 months	Arm A: n° of patients completing EPP: 22 (88%). Post-operative mortality rate: 4.5%. Arm B: CT completion rate 96.3%; Post-operative mortality rate: 0%.

CT: chemotherapy; EPP: extended pleurectomy decortication; EFS: event-free survival; HIOC: Hyperthermic IntraOperative Chemotherapy; ITT: intention to treat; OS: Overall Survival; pCR rate: pathological complete response; P/D: pleurectomy decortication; PFS: Progression-Free Survival; QoL: quality of life; RT: radiotherapy; RR: Response Rate; success of treatment, which is defined as a patient who received the full protocol treatment within the defined time-frames, and was still alive 90 days after the end of protocol treatment without progression or evidence of grade 3–4 toxicity.

**Table 3:** Immunotherapy/cancer vaccine and ongoing trials evaluating therapeutic strategies in peri-operative setting.

Immunotherapy and Cancer Vaccines						
Study Reference	Phase	Agent	Intervention	Patients N°	Primary Endpoint	Secondary Endpoint
**NCT02592551** (2023) [11]	Phase II	Durvalumab ± Tremelimumab	Cohort 1: pre-operative (1–6 weeks) single durvalumab; Cohort 2: pre-operative (1–6 weeks) single durvalumab + tremelimumab; Cohort 3: placebo; followed by surgery	24	No differences CD8/Treg ratio among the cohorts	Increased PD-L1 expression on EC after both single and combination ICI, and on M1-TAMs after combination ICI; mDFS: 8.4 mo (single ICI) vs. NR (dual ICI) [*p* = 0.009]; mOS: 14.0 mo (single ICI) vs. NR (dual ICI) [*p* = 0.040]
**Zaudere M. et al.** (2017) [62]	Phase II	Galinpepimut-S, WT-1 Analog Peptide Vaccine	Adjuvant galinpepimut-S plus GM-CSF and Montanide vs. GM-CSF and Montanide alone after surgery and another treatment modality	41	1/year PFS rate: 33% (experimental arm) vs. 45% (control arm)	\
**Study**	**Phase**	**Agent**	**Intervention**	**Setting**	**Primary endpoint**	**Status**
**Ongoing clinical trials on Chemotherapy**						
**NCT02436733** [63]	II	Pemetrexed + Cisplatin	Arm A—immediate P/D followed by adjuvant pemetrexed + cisplatin for non-progressing patients Arm B—Neoadjuvant pemetrexed + cisplatin followed by P/D, for non-progressing patients.	Adjuvant VS. Neoadjuvant	Rate of success to complete the full treatment	**Active, unknown status**
**NCT00715611** [61]	II	Pemetrexed + Cis-/carboplatin and IMRT	Neo-/adjuvant platin and pemetrexed proceeded or followed by P/D, and subsequent IMRT	Neo-/adjuvant + adjuvant IMRT	number of patients ≥ grade 3 pneumonitis	**Active, not recruiting**
**Ongoing clinical trials on radiotherapy**						
**NCT04028570** (SMARTER) [34]	Not applicable	Neoadjuvant RT	Starting cohort: Background radiation 0 cGy + 2100 Gy boost radiation to a part of GTV; if no DLT background radiation dose is increased to 600 cGy and up to 1800 cGy followed by surgery	Neoadjuvant	Maximum tolerated dose /Aes	**Active, not recruiting**
**NCT05380713** (SMARTEST) [35]	II	Neoadjuvant sub-ablative RT and cyclophosphamide	Sub-ablative RT plus cyclophosphamide vs. sub-ablative RT alone followed by surgery and adjuvant immunotherapy	Neoadjuavant	CD8 TIL density / GTV	**Recruiting**
**NCT03269227** (MesoRT) [36]	Not applicable	Adjuvant RT	Accelerated hypofractionation with Tomotherapy (30 Gy in 5 daily fr) with an internal increasing inhomogenous dose of up to 37.5–40 Gy for GTV	Adjuvant	AEs	**Unknown status**
**Ongoing clinical trials on Immune Checkpoint Inhibitors**						
**NCT03760575** [64]	I	Pembrolizumab	Neoadjuvant pembrolizumab followed by P/D and adjuvant chemo-IO and IO maintenance	Neoadjuvant	AEs	**Recruiting**
**NCT04162015** [65]	I	Nivolumab	Neoadjuvant nivolumab and platinum-based chemotherapy	Neoadjuvant	N of patients undergoing surgery	**Active, not recruiting**
**NCT03918252** [66]	I/II	Nivolumab ± ipilimumab	Neoadjuvant nivolumab ± ipilimumab for 3 cycles followed by surgery and adjuvant nivolumab for 12 months	Neoadjuvant	AEs/N of patients undergoing surgery	**Active, not recruiting**
**NCT03228537** [67]	I	Atezolizumab	Neoadjuvant atezolizumab, cisplatin and pemetrexed followed by surgery. Adjuvant atezolizymab for 12 months	Neoadjuvant	PFS/OS/ORR	**Active, not recruiting**
**NCT05647265** [68]	II	Nivolumab + ipilimumab	Neoadjuvant nivolumab + ipilimumab in sarcomatoid (>50%) mesothelioma	Neoadjuvant/adjuvant	Surgery rate/PFS	**Recruiting**
**NCT06155279** (CHIMERA) [69]	II	Pembrolizumab + Cisplatin/Carboplatin + Pemetrexed	Neoadjuvant pembrolizumab, cisplatin/carboplatin and pemetrexed, followed by surgery and adjuvant pembrolizumab for 12 months	Neoadjuvant/adjuvant	pCR	**Recruiting**
**NCT05932199** [70]	Ib/IIa	Durvalumab+ tremelimumab ± Cisplatin/Carboplatin + Pemetrexed	Neoadjuvant durvalumab + tremelimumab ± Cisplatin/Carboplatin + Pemetrexed for 3 cycles followed by surgery and adjuvant durvalumab for 12 months	Neoadjuvant/adjuvant	1 year-RFS	**Recruiting**
**NCT04996017** (AtezoMeso) [71]	III	Atezolizumab	Adjuvant atezolizumab/placebo in resected PM patients	Adjuvant	DFS	**Recruiting**
**Ongoing clinical trials on cancer vaccines**						
**NCT05304208** (ENSURE) [72]	I	DC therapy	Pre-operative DC therapy after NAC in resectable PM	Perioperative	Feasibility	**Recruiting**

AEs: adverse Events; DC: Dendritic Cells; DFS: Disease-Free Survival; DLT: Dose Limiting Toxicities; Fr: Fraction; GM-CSF: Granulocyte Macrophage Colony Stimulating Factor; GTV: Gross Tumor Volume; Gy: Gray; ICI: Immune Checkpoint Inhibitors; IMRT: Intensity-modulated radiotherapy; IO: Immunotherapy; NAC: Neoadjuvant Chemotherapy; ORR: Objective Response Rate; OS: Overall Survival; P/D: pleurectomy decortication; pCR: pathological complete response; PFS: Progression-Free Survival; RFS: Relapse-Free Survival; TAM: Tumor Associated Macrophages; TIL: Tumor-Infiltrating Lymphocytes.

## Data Availability

No new data were created or analyzed in this study.

## References

[1] Brims F. (2021). Epidemiology and Clinical Aspects of Malignant Pleural Mesothelioma. Cancers.

[2] Bovolato P., Casadio C., Billè A., Ardissone F., Santambrogio L., Ratto G.B., Garofalo G., Bedini A.V., Garassino M., Porcu L. (2014). Does Surgery Improve Survival of Patients with Malignant Pleural Mesothelioma?: A Multicenter Retrospective Analysis of 1365 Consecutive Patients. J. Thorac. Oncol..

[3] (2025). National Comprehensive Cancer Network (NCCN). Mesothelioma: Pleural.

[4] Scherpereel A., Opitz I., Berghmans T., Psallidas I., Glatzer M., Rigau D., Astoul P., Bölükbas S., Boyd J., Coolen J. (2020). ERS/ESTS/EACTS/ESTRO Guidelines for the Management of Malignant Pleural Mesothelioma. Eur. Respir. J..

[5] Popat S., Baas P., Faivre-Finn C., Girard N., Nicholson A.G., Nowak A.K., Opitz I., Scherpereel A., Reck M. (2022). Malignant Pleural Mesothelioma: ESMO Clinical Practice Guidelines for Diagnosis, Treatment and Follow-Up. Ann. Oncol..

[6] Treasure T., Lang-Lazdunski L., Waller D., Bliss J.M., Tan C., Entwisle J., Snee M., O’Brien M., Thomas G., Senan S. (2011). Extra-Pleural Pneumonectomy versus No Extra-Pleural Pneumonectomy for Patients with Malignant Pleural Mesothelioma: Clinical Outcomes of the Mesothelioma and Radical Surgery (MARS) Randomised Feasibility Study. Lancet Oncol..

[7] Lim E., Waller D., Lau K., Steele J., Pope A., Ali C., Bilancia R., Keni M., Popat S., O’Brien M. (2023). MARS 2: Extended Pleurectomy Decortication and Chemotherapy for Pleural Mesothelioma.

[8] Raskin J., Surmont V., Cornelissen R., Baas P., Van Schil P.E.Y., Van Meerbeeck J.P. (2018). A Randomized Phase II Study of Pleurectomy/Decortication Preceded or Followed by (Neo-)Adjuvant Chemotherapy in Patients with Early Stage Malignant Pleural Mesothelioma (EORTC 1205). Transl. Lung Cancer Res..

[9] Lim E., Opitz I., Woodard G., Bueno R., De Perrot M., Flores R., Gill R., Jablons D., Pass H. (2025). A Perspective on the MARS2 Trial. J. Thorac. Oncol..

[10] Kanayama M., Mori M., Matsumiya H., Taira A., Shinohara S., Takenaka M., Kuroda K., Tanaka F. (2022). Surgical Strategy for Malignant Pleural Mesothelioma: The Superiority of Pleurectomy/Decortication. Surg. Today.

[11] Lee H.-S., Jang H.-J., Ramineni M., Wang D.Y., Ramos D., Choi J.M., Splawn T., Espinoza M., Almarez M., Hosey L. (2023). A Phase II Window of Opportunity Study of Neoadjuvant PD-L1 versus PD-L1 plus CTLA-4 Blockade for Patients with Malignant Pleural Mesothelioma. Clin. Cancer Res..

[12] Nelson D.B., Rice D.C., Niu J., Atay S., Vaporciyan A.A., Antonoff M., Hofstetter W.L., Walsh G.L., Swisher S.G., Roth J.A. (2017). Long-Term Survival Outcomes of Cancer-Directed Surgery for Malignant Pleural Mesothelioma: Propensity Score Matching Analysis. J. Clin. Oncol..

[13] Spaggiari L., Marulli G., Bovolato P., Alloisio M., Pagan V., Oliaro A., Ratto G.B., Facciolo F., Sacco R., Brambilla D. (2014). Extrapleural Pneumonectomy for Malignant Mesothelioma: An Italian Multicenter Retrospective Study. Ann. Thorac. Surg..

[14] Zhou N., Rice D.C., Tsao A.S., Lee P.P., Haymaker C.L., Corsini E.M., Antonoff M.B., Hofstetter W.L., Rajaram R., Roth J.A. (2022). Extrapleural Pneumonectomy Versus Pleurectomy/Decortication for Malignant Pleural Mesothelioma. Ann. Thorac. Surg..

[15] Casiraghi M., Maisonneuve P., Brambilla D., Solli P., Galetta D., Petrella F., Piperno G., De Marinis F., Spaggiari L. (2017). Induction Chemotherapy, Extrapleural Pneumonectomy and Adjuvant Radiotherapy for Malignant Pleural Mesothelioma. Eur. J. Cardiothorac. Surg..

[16] Krug L.M., Pass H.I., Rusch V.W., Kindler H.L., Sugarbaker D.J., Rosenzweig K.E., Flores R., Friedberg J.S., Pisters K., Monberg M. (2009). Multicenter Phase II Trial of Neoadjuvant Pemetrexed Plus Cisplatin Followed by Extrapleural Pneumonectomy and Radiation for Malignant Pleural Mesothelioma. J. Clin. Oncol..

[17] Weder W., Stahel R.A., Bernhard J., Bodis S., Vogt P., Ballabeni P., Lardinois D., Betticher D., Schmid R., Stupp R. (2007). Multicenter Trial of Neo-Adjuvant Chemotherapy Followed by Extrapleural Pneumonectomy in Malignant Pleural Mesothelioma. Ann. Oncol..

[18] Marulli G., Faccioli E., Bellini A., Mammana M., Rea F. (2017). Induction Chemotherapy vs Post-Operative Adjuvant Therapy for Malignant Pleural Mesothelioma. Expert Rev. Respir. Med..

[19] Van Schil P.E., Baas P., Gaafar R., Maat A.P., Van De Pol M., Hasan B., Klomp H.M., Abdelrahman A.M., Welch J., Van Meerbeeck J.P. (2010). Trimodality Therapy for Malignant Pleural Mesothelioma: Results from an EORTC Phase II Multicentre Trial. Eur. Respir. J..

[20] Rea F., Favaretto A., Marulli G., Spaggiari L., De Pas M., Ceribelli A., Paccagnella A., Crivellari A., Russo F., Kazeem G. (2013). Phase II Trial of Neoadjuvant Pemetrexed plus Cisplatin Followed by Surgery and Radiation in the Treatment of Pleural Mesothelioma. BMC Cancer.

[21] Trovo M., Relevant A., Polesel J., Muraro E., Barresi L., Drigo A., Baresic T., Bearz A., Fanetti G., Del Conte A. (2021). Radical Hemithoracic Radiotherapy Versus Palliative Radiotherapy in Non-Metastatic Malignant Pleural Mesothelioma: Results from a Phase 3 Randomized Clinical Trial. Int. J. Radiat. Oncol. Biol. Phys..

[22] Thompson A.B., Quinn T.J., Siddiqui Z.A., Almahariq M.F., Grills I.S., Stevens C.W. (2020). Addition of Radiotherapy to Surgery and Chemotherapy Improves Survival in Localized Malignant Pleural Mesothelioma: A Surveillance, Epidemiology, and End Results (SEER) Study. Lung Cancer.

[23] Lian L., Lei H., Cheng S., Zheng R., Yao H., Chen J., Chen T. (2023). Survival Benefit after Radiotherapy for Patients with Malignant Pleural Mesothelioma: A Propensity Score—Matched Study. MedComm.

[24] Nelson D.B., Rice D.C., Mitchell K.G., Tsao A.S., Vaporciyan A.A., Antonoff M.B., Hofstetter W.L., Walsh G.L., Swisher S.G., Roth J.A. (2019). Defining the Role of Adjuvant Radiotherapy for Malignant Pleural Mesothelioma: A Propensity-Matched Landmark Analysis of the National Cancer Database. J. Thorac. Dis..

[25] Thieke C., Nicolay N.H., Sterzing F., Hoffmann H., Roeder F., Safi S., Debus J., Huber P.E. (2015). Long-Term Results in Malignant Pleural Mesothelioma Treated with Neoadjuvant Chemotherapy, Extrapleural Pneumonectomy and Intensity-Modulated Radiotherapy. Radiat. Oncol..

[26] Chance W.W., Rice D.C., Allen P.K., Tsao A.S., Fontanilla H.P., Liao Z., Chang J.Y., Tang C., Pan H.Y., Welsh J.W. (2015). Hemithoracic Intensity Modulated Radiation Therapy After Pleurectomy/Decortication for Malignant Pleural Mesothelioma: Toxicity, Patterns of Failure, and a Matched Survival Analysis. Int. J. Radiat. Oncol..

[27] Raman V., Voigt S.L., Jawitz O.K., Farrow N.E., Rhodin K.E., Yang C.-F.J., Tong B.C., D’Amico T.A., Harpole D.H. (2023). The Impact of Adjuvant Hemithoracic Radiation on Outcomes in Patients with Stage I-III Malignant Pleural Mesothelioma: A Dual Registry Analysis. Ann. Surg..

[28] Thompson M.R., Dumane V.A., Lazarev S.A., Zia Y., Rosenzweig K.E. (2019). Dosimetric Correlates of Pulmonary Toxicity in Patients with Malignant Pleural Mesothelioma Receiving Radiation Therapy to the Intact Lungs. Pract. Radiat. Oncol..

[29] Gomez D.R., Rimner A., Simone C.B., Cho B.C.J., De Perrot M., Adjei A.A., Bueno R., Gill R.R., Harpole D.H., Hesdorffer M. (2019). The Use of Radiation Therapy for the Treatment of Malignant Pleural Mesothelioma: Expert Opinion from the National Cancer Institute Thoracic Malignancy Steering Committee, International Association for the Study of Lung Cancer, and Mesothelioma Applied Research Foundation. J. Thorac. Oncol..

[30] Stahel R.A., Riesterer O., Xyrafas A., Opitz I., Beyeler M., Ochsenbein A., Früh M., Cathomas R., Nackaerts K., Peters S. (2015). Neoadjuvant Chemotherapy and Extrapleural Pneumonectomy of Malignant Pleural Mesothelioma with or without Hemithoracic Radiotherapy (SAKK 17/04): A Randomised, International, Multicentre Phase 2 Trial. Lancet Oncol..

[31] Riesterer O., Ciernik I.F., Stahel R.A., Xyrafas A., Aebersold D.M., Plasswilm L., Mahmut Ozsahin E., Zwahlen D.R., Nackaerts K., Zimmermann F. (2019). Pattern of Failure after Adjuvant Radiotherapy Following Extrapleural Pneumonectomy of Pleural Mesothelioma in the SAKK 17/04 Trial. Radiother. Oncol..

[32] Cho B.C.J., Donahoe L., Bradbury P.A., Leighl N., Keshavjee S., Hope A., Pal P., Cabanero M., Czarnecka K., McRae K. (2021). Surgery for Malignant Pleural Mesothelioma after Radiotherapy (SMART): Final Results from a Single-Centre, Phase 2 Trial. Lancet Oncol..

[33] Rimner A., Zauderer M.G., Gomez D.R., Adusumilli P.S., Parhar P.K., Wu A.J., Woo K.M., Shen R., Ginsberg M.S., Yorke E.D. (2016). Phase II Study of Hemithoracic Intensity-Modulated Pleural Radiation Therapy (IMPRINT) as Part of Lung-Sparing Multimodality Therapy in Patients with Malignant Pleural Mesothelioma. J. Clin. Oncol..

[34] National Cancer Institute (NCI) A Feasibility Study Evaluating Surgery for Mesothelioma After Radiation Therapy Using Extensive Pleural Resection (SMARTER). (NCT04028570). NCT04028570.

[35] National Cancer Institute (NCI) SMARTEST Trial. (NCT05380713). NCT05380713.

[36] National Cancer Institute (NCI) Accelerated Hypofractionated Radiotherapy in the Treatment of Malignant Pleural Mesothelioma (MesoRT). (NCT03269227). NCT03269227.

[37] Parisi E., Arpa D., Ghigi G., Fabbri L., Foca F., Tontini L., Neri E., Pieri M., Cima S., Burgio M.A. (2023). Malignant Pleural Mesothelioma: Preliminary Toxicity Results of Adjuvant Radiotherapy Hypofractionation in a Prospective Trial (MESO-RT). Cancers.

[38] Mohan R., Grosshans D. (2017). Proton Therapy—Present and Future. Adv. Drug Deliv. Rev..

[39] Patel N.V., Yu N.Y., Koroulakis A., Diwanji T., Sawant A., Sio T.T., Mohindra P. (2021). Proton Therapy for Thoracic Malignancies: A Review of Oncologic Outcomes. Expert Rev. Anticancer Ther..

[40] Lee H., Zeng J., Bowen S.R., Rengan R. (2017). Proton Therapy for Malignant Pleural Mesothelioma: A Three Case Series Describing the Clinical and Dosimetric Advantages of Proton-Based Therapy. Cureus.

[41] Pan H.Y., Jiang S., Sutton J., Liao Z., Chance W.W., Frank S.J., Zhu X.R., Li H., Fontanilla H.P., Zhang X. (2015). Early Experience with Intensity Modulated Proton Therapy for Lung-Intact Mesothelioma: A Case Series. Pract. Radiat. Oncol..

[42] Zeng J., Badiyan S.N., Garces Y.I., Wong T., Zhang X., Simone C.B., Chang J.Y., Knopf A.C., Mori S., Iwata H. (2021). Consensus Statement on Proton Therapy in Mesothelioma. Pract. Radiat. Oncol..

[43] Pagan V., Ceron L., Paccagnella A., Pizzi G. (2006). 5-Year Prospective Results of Trimodality Treatment for Malignant Pleural Mesothelioma. J. Cardiovasc. Surg..

[44] Lang-Lazdunski L., Bille A., Lal R., Cane P., McLean E., Landau D., Steele J., Spicer J. (2012). Pleurectomy/Decortication Is Superior to Extrapleural Pneumonectomy in the Multimodality Management of Patients with Malignant Pleural Mesothelioma. J. Thorac. Oncol..

[45] Opitz I., Kestenholz P., Lardinois D., Müller M., Rousson V., Schneiter D., Stahel R., Weder W. (2006). Incidence and Management of Complications after Neoadjuvant Chemotherapy Followed by Extrapleural Pneumonectomy for Malignant Pleural Mesothelioma. Eur. J. Cardiothorac. Surg..

[46] Flores R.M., Krug L.M., Rosenzweig K.E., Venkatraman E., Vincent A., Heelan R., Akhurst T., Rusch V.W. (2006). Induction Chemotherapy, Extrapleural Pneumonectomy, and Postoperative High-Dose Radiotherapy for Locally Advanced Malignant Pleural Mesothelioma: A Phase II Trial. J. Thorac. Oncol..

[47] Buduhan G., Menon S., Aye R., Louie B., Mehta V., Vallières E. (2009). Trimodality Therapy for Malignant Pleural Mesothelioma. Ann. Thorac. Surg..

[48] De Perrot M., Feld R., Cho B.C.J., Bezjak A., Anraku M., Burkes R., Roberts H., Tsao M.S., Leighl N., Keshavjee S. (2009). Trimodality Therapy with Induction Chemotherapy Followed by Extrapleural Pneumonectomy and Adjuvant High-Dose Hemithoracic Radiation for Malignant Pleural Mesothelioma. J. Clin. Oncol..

[49] Pasello G., Marulli G., Polo V., Breda C., Bonanno L., Loreggian L., Rea F., Favaretto A. (2012). Pemetrexed plus carboplatin or cisplatin as neoadjuvant treatment of operable malignant pleural mesothelioma (MPM). Anticancer Res..

[50] Lauk O., Hoda M.A., De Perrot M., Friess M., Klikovits T., Klepetko W., Keshavjee S., Weder W., Opitz I. (2014). Extrapleural Pneumonectomy After Induction Chemotherapy: Perioperative Outcome in 251 Mesothelioma Patients from Three High-Volume Institutions. Ann. Thorac. Surg..

[51] Hasegawa S., Okada M., Tanaka F., Yamanaka T., Soejima T., Kamikonya N., Tsujimura T., Fukuoka K., Yokoi K., Nakano T. (2016). Trimodality Strategy for Treating Malignant Pleural Mesothelioma: Results of a Feasibility Study of Induction Pemetrexed plus Cisplatin Followed by Extrapleural Pneumonectomy and Postoperative Hemithoracic Radiation (Japan Mesothelioma Interest Group 0601 Trial). Int. J. Clin. Oncol..

[52] Sugarbaker D.J., Flores R.M., Jaklitsch M.T., Richards W.G., Strauss G.M., Corson J.M., DeCamp M.M., Swanson S.J., Bueno R., Lukanich J.M. (1999). Resection Margins, Extrapleural Nodal Status, and Cell Type Determine Postoperative Long-Term Survival in Trimodality Therapy of Malignant Pleural Mesothelioma: Results in 183 Patients. J. Thorac. Cardiovasc. Surg..

[53] Luckraz H., Rahman M., Patel N., Szafranek A., Gibbs A.R., Butchart E.G. (2010). Three Decades of Experience in the Surgical Multi-Modality Management of Pleural Mesothelioma. Eur. J. Cardiothorac. Surg..

[54] Borasio P., Berruti A., Billé A., Lausi P., Levra M.G., Giardino R., Ardissone F. (2008). Malignant Pleural Mesothelioma: Clinicopathologic and Survival Characteristics in a Consecutive Series of 394 Patients. Eur. J. Cardiothorac. Surg..

[55] Ambrogi V., Baldi A., Schillaci O., Mineo T.C. (2012). Clinical Impact of Extrapleural Pneumonectomy for Malignant Pleural Mesothelioma. Ann. Surg. Oncol..

[56] Bölükbas S., Manegold C., Eberlein M., Bergmann T., Fisseler-Eckhoff A., Schirren J. (2011). Survival after Trimodality Therapy for Malignant Pleural Mesothelioma: Radical Pleurectomy, Chemotherapy with Cisplatin/Pemetrexed and Radiotherapy. Lung Cancer.

[57] Batirel H.F., Metintas M., Caglar H.B., Yildizeli B., Lacin T., Bostanci K., Akgul A.G., Evman S., Yuksel M. (2008). Trimodality Treatment of Malignant Pleural Mesothelioma. J. Thorac. Oncol..

[58] Verma V., Ahern C.A., Berlind C.G., Lindsay W.D., Grover S., Friedberg J.S., Simone C.B. (2019). Treatment of Malignant Pleural Mesothelioma with Chemotherapy Preceding versus after Surgical Resection. J. Thorac. Cardiovasc. Surg..

[59] Sharkey A.J., O’Byrne K.J., Nakas A., Tenconi S., Fennell D.A., Waller D.A. (2016). How Does the Timing of Chemotherapy Affect Outcome Following Radical Surgery for Malignant Pleural Mesothelioma?. Lung Cancer.

[60] Marulli G., Rea F., Nicotra S., Favaretto A.G., Perissinotto E., Chizzolini M., Vianello A., Braccioni F. (2010). Effect of Induction Chemotherapy on Lung Function and Exercise Capacity in Patients Affected by Malignant Pleural Mesothelioma. Eur. J. Cardiothorac. Surg..

[61] National Cancer Institute (NCI) Pleurectomy/Decortication (Neo) Adjuvant Chemotherapy and Intensity Modulated Radiation Therapy to the Pleura in Patients with Locally Advanced Malignant Pleural Mesothelioma. (NCT00715611). NCT00715611.

[62] Zauderer M.G., Tsao A.S., Dao T., Panageas K., Lai W.V., Rimner A., Rusch V.W., Adusumilli P.S., Ginsberg M.S., Gomez D. (2017). A Randomized Phase II Trial of Adjuvant Galinpepimut-S, WT-1 Analogue Peptide Vaccine, After Multimodality Therapy for Patients with Malignant Pleural Mesothelioma. Clin. Cancer Res..

[63] National Cancer Institute (NCI) Pleurectomy/Decortication (P/D) Preceded or Followed by Chemotherapy in Patients with Early Stage MPM. (NCT02436733). NCT02436733.

[64] National Cancer Institute (NCI) Pembrolizumab in Combination with Chemotherapy and Image-Guided Surgery for Malignant Pleural Mesothelioma (MPM). (NCT03760575). NCT03760575.

[65] National Cancer Institute (NCI) A Study of Nivolumab and Chemotherapy Followed by Surgery for Mesothelioma. (NCT04162015). NCT04162015.

[66] National Cancer Institute (NCI) Neoadjuvant Immune Checkpoint Blockade in Resectable Malignant Pleural Mesothelioma. (NCT03918252). NCT03918252.

[67] National Cancer Institute (NCI) Atezolizumab, Pemetrexed Disodium, Cisplatin, and Surgery With or Without Radiation Therapy in Treating Patients with Stage I-III Pleural Malignant Mesothelioma. (NCT03228537). NCT03228537.

[68] National Cancer Institute (NCI) Testing the Addition of Immunotherapy Before Surgery for Patients with Sarcomatoid Mesothelioma. (NCT05647265). NCT05647265.

[69] National Cancer Institute (NCI) Induction Chemo+Immunotherapy in Resectable Epithelioid and Biphasic Pleural Mesothelioma (CHIMERA Study) (CHIMERA). (NCT06155279). NCT06155279.

[70] National Cancer Institute (NCI) Neoadjuvant Durvalumab and Tremelimumab with and Without Chemotherapy for Mesothelioma. (NCT05932199). NCT05932199.

[71] National Cancer Institute (NCI) Atezolizumab Versus Placebo for the Adjuvant Treatment of Malignant Pleural Mesothelioma (Atezomeso) (AtezoMeso). (NCT04996017). NCT04996017.

[72] Giraudo M., Jackson Z., Das I., Abiona O., Wald D. (2024). Chimeric Antigen Receptor (CAR)-T Cell Therapy for Non-Hodgkin’s Lymphoma. Pathog. Immun..

[73] Okubo K., Ishibashi H., Wakejima R., Baba S., Asakawa A., Seshima H. (2023). Extended Pleurectomy/Decortication and Hyperthermic Intraoperative Intrapleural Cisplatin Perfusion for Malignant Pleural Mesothelioma. JTCVS Open.

[74] Bongiolatti S., Mazzoni F., Salimbene O., Caliman E., Ammatuna C., Comin C.E., Antonuzzo L., Voltolini L. (2021). Induction Chemotherapy Followed by Pleurectomy Decortication and Hyperthermic Intraoperative Chemotherapy (HITHOC) for Early-Stage Epitheliod Malignant Pleural Mesothelioma—A Prospective Report. J. Clin. Med..

[75] Elsayed H.H., Elanany M.E.E., ElSayegh M.T., Hassaballa A.S., Abdel-gayed M. (2025). Hyperthermic Intrathoracic Chemotherapy in Patients with Malignant Pleural Mesothelioma after Cytoreductive Surgical Procedures: A Systematic Review. World J. Surg. Oncol..

[76] Van Sandick J.W., Kappers I., Baas P., Haas R.L., Klomp H.M. (2008). Surgical Treatment in the Management of Malignant Pleural Mesothelioma: A Single Institution’s Experience. Ann. Surg. Oncol..

[77] Sugarbaker D.J., Gill R.R., Yeap B.Y., Wolf A.S., DaSilva M.C., Baldini E.H., Bueno R., Richards W.G. (2013). Hyperthermic Intraoperative Pleural Cisplatin Chemotherapy Extends Interval to Recurrence and Survival among Low-Risk Patients with Malignant Pleural Mesothelioma Undergoing Surgical Macroscopic Complete Resection. J. Thorac. Cardiovasc. Surg..

[78] Elsayed H.H., Sharkawy H.Y., Ahmed M.A., Abdel-Gayed M., Eldewer M. (2024). Effect of Intraoperative Hyperthermic Intrathoracic Chemotherapy after Pleurectomy Decortication for Treatment of Malignant Pleural Mesothelioma: A Comparative Study. Updat. Surg..

[79] Harber J., Kamata T., Pritchard C., Fennell D. (2021). Matter of TIME: The Tumor-Immune Microenvironment of Mesothelioma and Implications for Checkpoint Blockade Efficacy. J. Immunother. Cancer.

[80] Marcq E., Siozopoulou V., De Waele J., Van Audenaerde J., Zwaenepoel K., Santermans E., Hens N., Pauwels P., Van Meerbeeck J.P., Smits E.L.J. (2017). Prognostic and Predictive Aspects of the Tumor Immune Microenvironment and Immune Checkpoints in Malignant Pleural Mesothelioma. OncoImmunology.

[81] Brcic L., Klikovits T., Megyesfalvi Z., Mosleh B., Sinn K., Hritcu R., Laszlo V., Cufer T., Rozman A., Kern I. (2021). Prognostic Impact of PD-1 and PD-L1 Expression in Malignant Pleural Mesothelioma: An International Multicenter Study. Transl. Lung Cancer Res..

[82] Lee H.-S., Hamaji M., Palivela N., Jang H.-J., Splawn T., Ramos D., Lee A.K., Raghuram A.C., Ramineni M., Amos C.I. (2021). Prognostic Role of Programmed Cell Death 1 Ligand 1 in Resectable Pleural Mesothelioma. Ann. Thorac. Surg..

[83] Baas P., Scherpereel A., Nowak A.K., Fujimoto N., Peters S., Tsao A.S., Mansfield A.S., Popat S., Jahan T., Antonia S. (2021). First-Line Nivolumab plus Ipilimumab in Unresectable Malignant Pleural Mesothelioma (CheckMate 743): A Multicentre, Randomised, Open-Label, Phase 3 Trial. Lancet.

[84] Chu Q., Perrone F., Greillier L., Tu W., Piccirillo M.C., Grosso F., Lo Russo G., Florescu M., Mencoboni M., Morabito A. (2023). Pembrolizumab plus Chemotherapy versus Chemotherapy in Untreated Advanced Pleural Mesothelioma in Canada, Italy, and France: A Phase 3, Open-Label, Randomised Controlled Trial. Lancet.

[85] Jin L., Gu W., Li X., Xie L., Wang L., Chen Z. (2020). PD-L1 and Prognosis in Patients with Malignant Pleural Mesothelioma: A Meta-Analysis and Bioinformatics Study. Ther. Adv. Med. Oncol..

[86] Pagano M., Alloisio M., Cappuzzo F., Facciolo F., Bironzo P., Pastorino U., Rea F., Santoro A., Zanelli F., Alberti G. (2022). Phase III Study with Atezolizumab versus Placebo in Patients with Malignant Pleural Mesothelioma after Pleurectomy/Decortication (AtezoMeso Study). J. Clin. Oncol..

[87] National Cancer Institute (NCI) Nivolumab with Chemotherapy in Pleural Mesothelioma After Surgery. (NCT04177953). NCT04177953.

[88] Shah R., Klotz L.V., Chung I., Feißt M., Schneider M.A., Riedel J., Bischoff H., Eichhorn M.E., Thomas M. (2021). A Phase II Trial of Nivolumab with Chemotherapy Followed by Maintenance Nivolumab in Patients with Pleural Mesothelioma After Surgery: The NICITA Study Protocol. Clin. Lung Cancer.

[89] Heymach J.V., Harpole D., Mitsudomi T., Taube J.M., Galffy G., Hochmair M., Winder T., Zukov R., Garbaos G., Gao S. (2023). Perioperative Durvalumab for Resectable Non–Small-Cell Lung Cancer. N. Engl. J. Med..

[90] Patel S.P., Othus M., Chen Y., Wright G.P., Yost K.J., Hyngstrom J.R., Hu-Lieskovan S., Lao C.D., Fecher L.A., Truong T.-G. (2023). Neoadjuvant–Adjuvant or Adjuvant-Only Pembrolizumab in Advanced Melanoma. N. Engl. J. Med..

[91] Forde P.M., Spicer J., Lu S., Provencio M., Mitsudomi T., Awad M.M., Felip E., Broderick S.R., Brahmer J.R., Swanson S.J. (2022). Neoadjuvant Nivolumab plus Chemotherapy in Resectable Lung Cancer. N. Engl. J. Med..

[92] Blank C.U., Rozeman E.A., Fanchi L.F., Sikorska K., Van De Wiel B., Kvistborg P., Krijgsman O., Van Den Braber M., Philips D., Broeks A. (2018). Neoadjuvant versus Adjuvant Ipilimumab plus Nivolumab in Macroscopic Stage III Melanoma. Nat. Med..

[93] Tsao A., Qian L., Cetnar J., Sepesi B., Gomez D., Wrangle J., Simon G., Mott F., Hall R., Santana-Davila R. (2021). OA13.01 S1619 A Trial of Neoadjuvant Cisplatin-Pemetrexed with Atezolizumab in Combination and Maintenance for Resectable Pleural Mesothelioma. J. Thorac. Oncol..

[94] National Cancer Institute (NCI) Using a Targeted Cancer Vaccine (Galinpepimut-S) with Immunotherapy (Nivolumab) in Mesothelioma. (NCT04040231). NCT04040231.

[95] National Cancer Institute (NCI) Poly-ICLC (Hiltonol^®^) Vaccine In Malignant Pleural Mesothelioma. (NCT04525859). NCT04525859.

[96] Fitzgerald B.G., Marron T.U., Sweeney R., Gomez J., Hall N., O’Grady D., Rolfo C., Veluswamy R., Doroshow D., Mandeli J. (2022). Abstract CT205: A Phase I/Ib Trial of Intratumoral Poly-ICLC in Resectable Malignant Pleural Mesothelioma. Cancer Res..

[97] Cornelissen R., Hegmans J.P.J.J., Maat A.P.W.M., Kaijen-Lambers M.E.H., Bezemer K., Hendriks R.W., Hoogsteden H.C., Aerts J.G.J.V. (2016). Extended Tumor Control after Dendritic Cell Vaccination with Low-Dose Cyclophosphamide as Adjuvant Treatment in Patients with Malignant Pleural Mesothelioma. Am. J. Respir. Crit. Care Med..

[98] National Cancer Institute (NCI) dENdritic Cell Therapy Combined with SURgEry in Mesothelioma (ENSURE). (NCT05304208). NCT05304208.

[99] Castelletti L., Yeo D., Van Zandwijk N., Rasko J.E.J. (2021). Anti-Mesothelin CAR T Cell Therapy for Malignant Mesothelioma. Biomark. Res..

[100] Haas A.R., Tanyi J.L., O’Hara M.H., Gladney W.L., Lacey S.F., Torigian D.A., Soulen M.C., Tian L., McGarvey M., Nelson A.M. (2019). Phase I Study of Lentiviral-Transduced Chimeric Antigen Receptor-Modified T Cells Recognizing Mesothelin in Advanced Solid Cancers. Mol. Ther..

[101] Adusumilli P.S., Zauderer M.G., Rivière I., Solomon S.B., Rusch V.W., O’Cearbhaill R.E., Zhu A., Cheema W., Chintala N.K., Halton E. (2021). A Phase I Trial of Regional Mesothelin-Targeted CAR T-Cell Therapy in Patients with Malignant Pleural Disease, in Combination with the Anti–PD-1 Agent Pembrolizumab. Cancer Discov..

[102] Marulli G., Breda C., Ratto G.B., Leoncini G., Alloisio M., Infante M., Luzzi L., Paladini P., Oliaro A., Ruffini E. (2017). Pleurectomy-decortication in malignant pleural mesothelioma: Are different surgical techniques associated with different outcomes? Results from a multicentre study. Eur. J. Cardiothorac. Surg..

